# Modified Johannesburg technique sets the standard – superior biomechanical stability in chest tube fixation

**DOI:** 10.1007/s00068-025-03071-7

**Published:** 2026-01-22

**Authors:** Johann Justus Fricke, Tobias Schöbel, Ric Meißner, Stefan Schleifenbaum, Christian Kleber, Isabella Metelmann, Sebastian Krämer

**Affiliations:** 1 Department of Orthopedic, Trauma, and Plastic Surgery, University of Leipzig, Liebigstraße 20, 04103 Leipzig, Germany; 2 ZESBO - Center for Research on Musculoskeletal Systems, Semmelweisstraße 14, 04103 Leipzig, Germany; 3 Fraunhofer Institute for Machine Tools and Forming Technology, Nöthnitzer Straße 44, 01187 Dresden, Germany; 4 Department of Visceral, Transplantation, Thoracic and Vascular Surgery, Leipzig University, Liebigstraße 20, 04013 Leipzig, Germany

**Keywords:** Chest tubes, Thoracic injuries, Suture, Biomechanics, Fixation techniques, Trauma, Emergency, Combat care

## Abstract

**Background:**

Chest tube dislodgement is a frequent and potentially life-threatening complication after thoracic trauma. Reliable fixation is essential, particularly in emergency and combat casualty care. Several suture techniques exist, but comparative biomechanical evidence is limited.

**Methods:**

This prospective biomechanical cadaveric study compared three fixation techniques—Purse String (PS), Roman Sandal (RS), and Modified Johannesburg (JO) technique —across three tube sizes (20, 24, 28 Charrière) and two materials (polyvinyl chloride (PVC) and silicone). A total of 360 tests were performed on porcine thoracic wall specimens under standardized conditions. Specimens were subjected to vertical load-to-failure testing with continuous measurement of force and elongation.

**Results:**

The Modified Johannesburg technique consistently demonstrated the highest pull-out strength across all tube sizes and materials, significantly outperforming both RS and PS (*p* < 0.0001). RS ranked second and showed significantly greater stability than PS in all conditions (*p* < 0.0001). Effect size analysis revealed large differences between JO and PS (*r* = 0.87) and between RS and PS (*r* = 0.50), and a medium effect between JO and RS (*r* = 0.36). PVC tubes provided significantly greater pull-out strength than silicone tubes (147.0 ± 54.2 N vs. 128.5 ± 42.2 N; *p* = 0.01071), although silicone tubes exhibited greater elongation prior to failure. Tube size had a modest influence on stability, with larger tubes tending toward higher pull-out strength, but pairwise differences did not reach significance. The predominant failure mechanism was suture rupture (78.9%), followed by tube rupture (11.1%), knot loosening (8.1%), and skin failure (1.9%).

**Conclusions:**

The Modified Johannesburg Technique offers superior biomechanical stability for chest tube fixation, regardless of tube size or material. This technique should be considered the preferred method in high-risk environments, including military and emergency care, to minimize dislodgement and enhance patient safety.

**Supplementary Information:**

The online version contains supplementary material available at 10.1007/s00068-025-03071-7.

## Background

Thoracic trauma remains one of the most common and potentially lethal injuries in both civilian and military populations. Non decompressed tension pneumothorax poses the most common definitive preventable traumatic death [1, 2]. Tube thoracostomy is the standard of care for pneumothorax and hemothorax, enabling rapid pleural decompression and hemodynamic stabilization. Despite its widespread use, complication rates approach 19% [3], with chest tube dislodgement being one of the most serious, especially in cases of a prolonged duration [4]. Complications in tube thoracostomy are associated, among other factors, with placement in the emergency department, insertion by emergency medicine physicians, and elevated body mass index [5]. Dislodgement rates reported in the literature range from 1.3% to 21% [3, 6] and may necessitate repeat tube placement, prolong hospitalization, or result in life-threatening events such as recurrent (tension) pneumothorax or hemorrhage [7–10]. Reliable fixation is therefore essential for both patient survival and operational safety.

Several fixation techniques have been described, including the Purse String (PS), Roman Sandal (RS), and Modified Johannesburg (JO) sutures [11–13]. Biomechanical studies indicate that the Modified Johannesburg technique, especially when using a size-1 silk suture, provides superior mechanical stability, with resistance forces up to threefold higher than those of the purse-string and nearly tenfold greater than commercial adhesive devices [14]. Techniques with multiple skin anchoring points also tend to be more robust [15]. The Roman Sandal offers a rapid and cosmetically favorable alternative, though its biomechanical performance is less well quantified [12] and might be insecure in clinical settings [13].

Given the dynamic stresses encountered during patient transport, resuscitation or combat operations, there is a critical need for evidence-based guidance on optimal chest tube fixation. This study evaluates three of the most common fixation techniques: Purse String, Roman Sandal, and Modified Johannesburg—across three tube sizes (20 Ch, 24 Ch, 28 Ch) and two materials (PVC, silicone) in a controlled porcine cadaver model. Load-to-failure testing and failure mode analysis were performed to generate practical recommendations, aiming to reduce complications and improve outcomes in trauma and acute care surgery.

We hypothesized that the Modified Johannesburg technique would yield higher breaking forces than the other two techniques, while the effects of tube size and material would be negligible.

## Methods

### Study design

This prospective interventional biomechanical cadaveric study evaluated chest tube fixation techniques using porcine thoracic wall specimens. A total of 360 experiments were conducted under standardized laboratory conditions to compare three fixation methods.

## Specimens and preparation

**Fig. 1:**
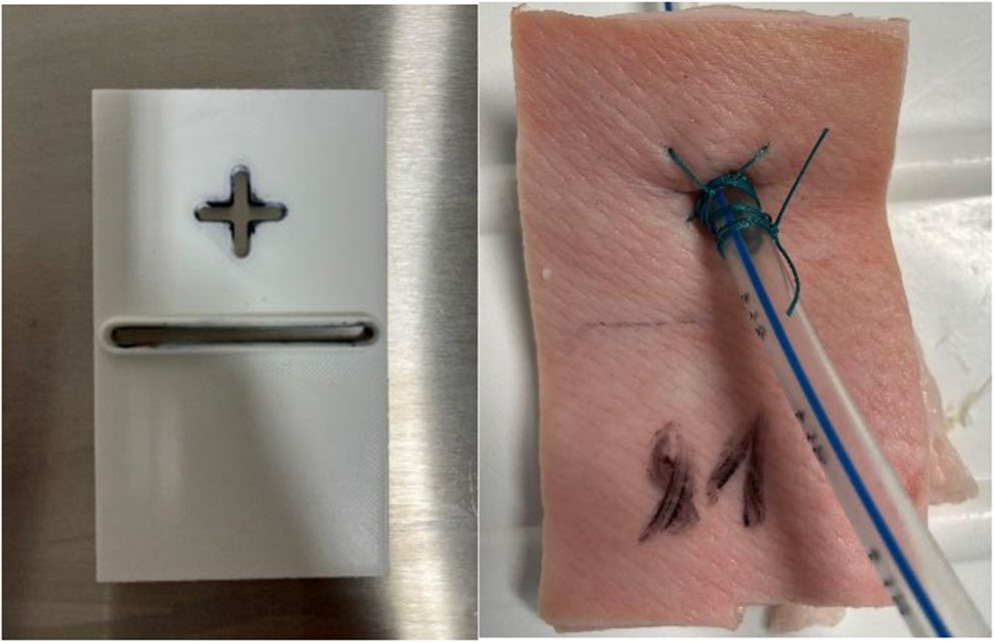
**A** Standardized Template for specimen preparation to allow **B** standardized marking of the inferior half ot the specimen an exact drain position

Fresh porcine thoracic wall samples were obtained either on the day of testing or one day prior. Specimens were stored under refrigeration at 4 °C until use. Rectangular Sect. (10 × 6 cm) with an average thickness of 1.5 cm were prepared from each thoracic wall, using a standardized template (Fig. 1). A central perforation was created for tube insertion. Specimens were marked at 5 cm: the lower mark defined the clamp position within the testing device, and the upper mark indicated the tube insertion site.

## Experimental groups

A total of 360 tests were conducted across 18 groups, defined by fixation technique, tube size, and tube material (Table 1).

**Table 1:** Configuration of the 18 groups, consting of fixation technique, tube size and tested material. Ch: charriére. PVC: Polyvinyl chloride

Fixation technique	Tube size (Ch)	Material tested
**Purse String (PS)**	28, 24, 20	PVC; Silicone
**Roman Sandal (RS)**	28, 24, 20	PVC; Silicone
**Modified Johannesburg (JO)**	28, 24, 20	PVC; Silicone

**Fig. 2:**
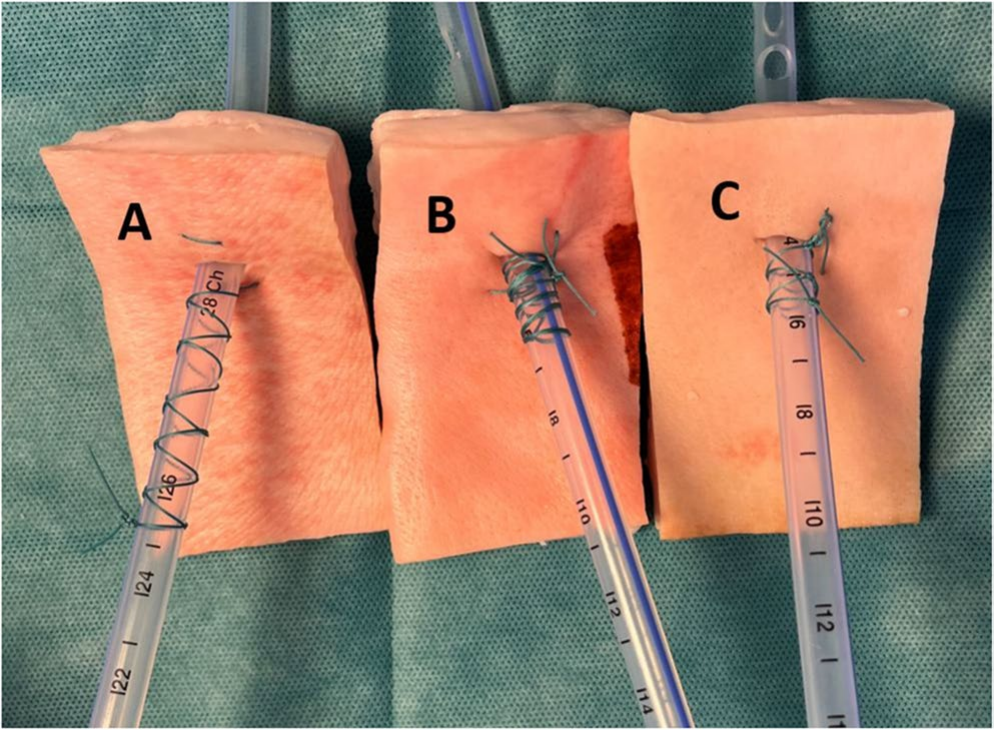
Fixation techniques compared: **A** Roman Sandal. **B** Modified Johannesburg. **C** Purse String

Fixations were performed by two experienced thoracic surgeons using non-absorbable 1 − 0 Mersilene sutures (Ethicon, Inc., Somerville, NJ, USA) and either PVC or silicone tubes (Primed Halberstadt Medizintechnik GmbH, Halberstedt, Germany) (Fig. 2). Specimens were labeled with alphanumeric codes representing tube size, material and suture technique The complete set of codes was randomized using the randomization service provided by random.org (Randomness and Integrity Services Ltd., Dublin, Ireland).

## Testing protocol

**Fig. 3:**
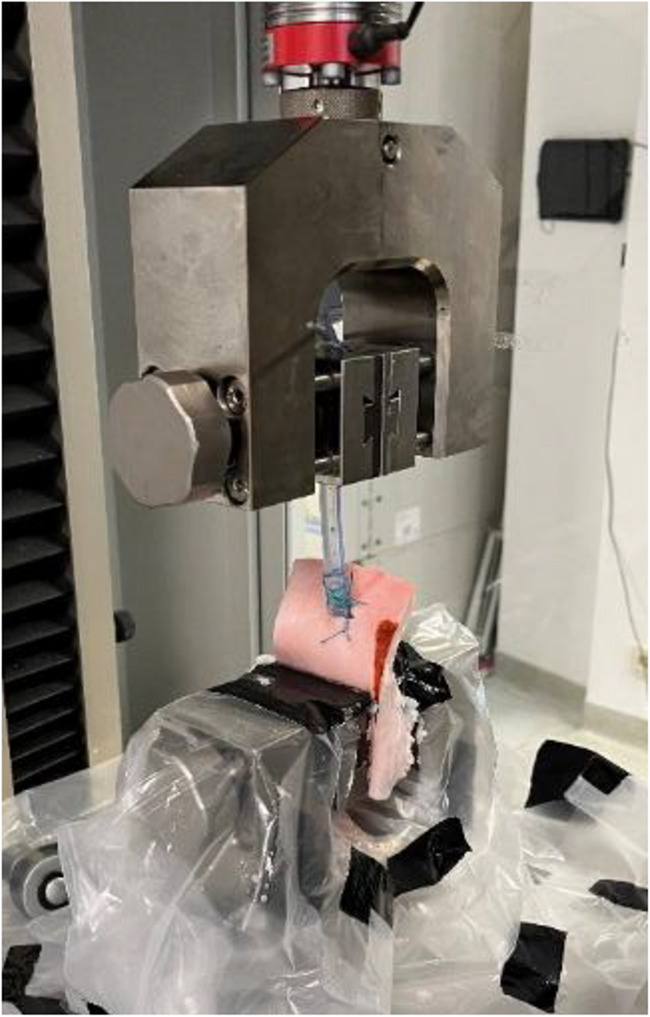
Test setup showing the specimen in uniaxial testing machine

Specimens were mounted in the lower clamps of a uniaxial testing machine (Allroundline Z10, Zwick/Roell GmbH & Co. KG, Ulm, Germany) with a 2.5 kN load cell. The inserted tube was secured at 7 cm distance from the upper clamp (Fig. 3). A frontal camera (Canon EOS 70D; Canon Inc., Tokio, Japan) was used for documentation. A vertical load-to-failure (LTF) test was performed under standardized conditions, continuously recording force (N) and crosshead travel (mm) until specimen failure. Prior to testing, a preload of 5 N was applied. The test was conducted at the maximum achievable crosshead speed of 1750 mm/min. A vertical load-to-failure (LTF) test was applied under standardized conditions, continuously recording force (in N) and elongation (in mm) until specimen failure. The failure mode was documented for each experiment.

### Statistical analysis

Statistical analyses were conducted in R (version 2025.09.0 + 387; R Core Team, Vienna, Austria). Data distribution was assessed using the Kolmogorov–Smirnov and Shapiro–Wilk tests. Group comparisons were performed with the Kruskal–Wallis test and Dunn’s post hoc test with Bonferroni correction or, where appropriate, the Mann–Whitney-U-test. Associations between categorical variables were analyzed with the Chi-square test. Effect sizes were reported as r = |z/√N|, and significance was set at *p* < 0.05.

## Results

The data did not follow a normal distribution, as indicated by both the Kolmogorov–Smirnov test (*p* = 0.001632) and the Shapiro–Wilk test (*p* < 0.0001).

## Effect of suture technique

**Fig. 4:**
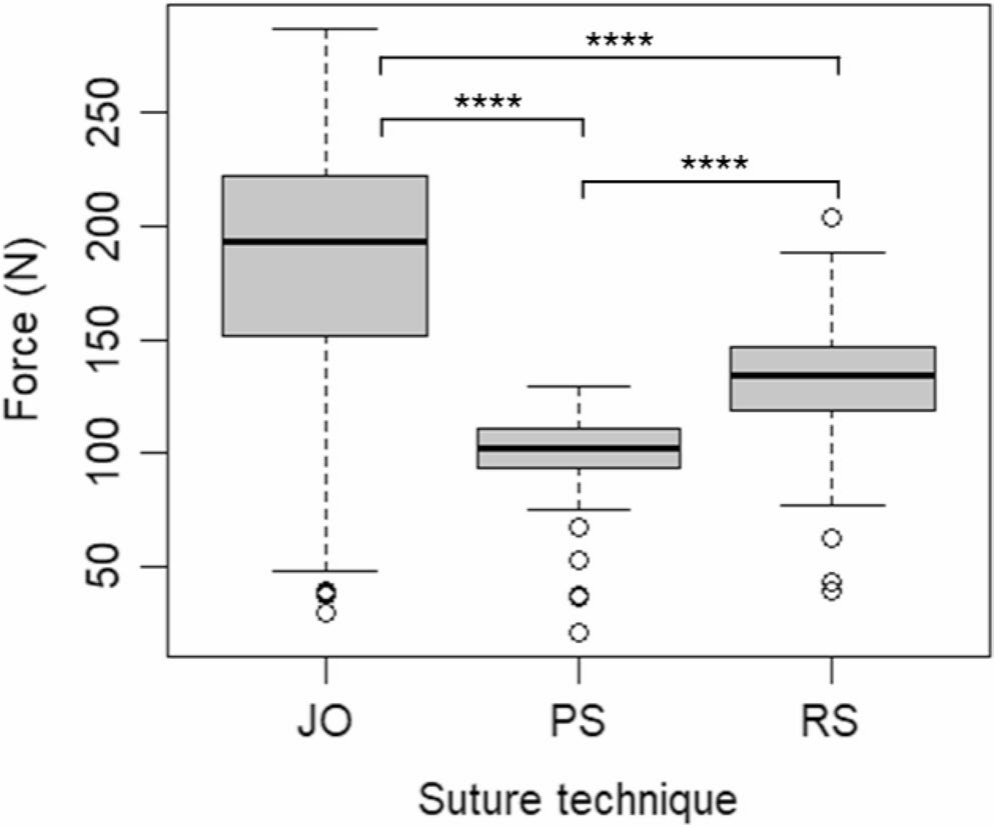
Boxplots of the breaking force in N (Newton) of three different suture techniques across all tube sizes and materials. JO (Modified Johannesburg), RS (Roman Sandal) and PS (Purse String), **** *p* < 0.0001

The Modified Johannesburg technique consistently demonstrated the highest pull-out strength across all tube sizes and materials (Table 2; Fig. 4) significantly outperforming both Roman Sandal (*p* < 0.0001) and purse-string (*p* < 0.0001) techniques. The Roman Sandal technique ranked second, showing significantly greater pull-out strength than the Purse String technique in all tested conditions (*p* < 0.0001). Effect sizes indicated a large effect between the Modified Johannesburg and Purse String techniques (*r* = 0.87), a large effect between the Roman Sandal and Purse String techniques (*r* = 0.50), and a medium effect between the Modified Johannesburg and Roman Sandal techniques (*r* = 0.36).

**Table 2:** Mean breaking forces in N (Newton) in load-to-failure testing for all groups. Ch: Charrière, PVC: Polyvinylchloride, ±: standard deviation

Technique	Roman Sandal			Purse-String			Modified Johannesburg		
Tube Size	20 Ch	24 Ch	28 Ch	20 Ch	24 Ch	28 Ch	20 Ch	24 Ch	28 Ch
**Silicone**	117.4 (± 33.8)	131.0 (± 19.4)	135.4 (± 21.8)	93.6 (± 15.0)	99.9 (± 18.1)	100.7 (± 21.5)	130.5 (± 35.5)	178.0 (± 32.8)	170.4 (± 62.3)
**PVC**	130.8 (± 22.5)	137.3 (± 22.2)	142.5 (± 19.2)	103.0 (± 11.8)	100.0 (± 17.5)	102.3 (± 11.7)	195.3 (± 60.2)	193.61 (± 58.3)	217.9 (± 42.9)

## Effect of material

**Fig. 5:**
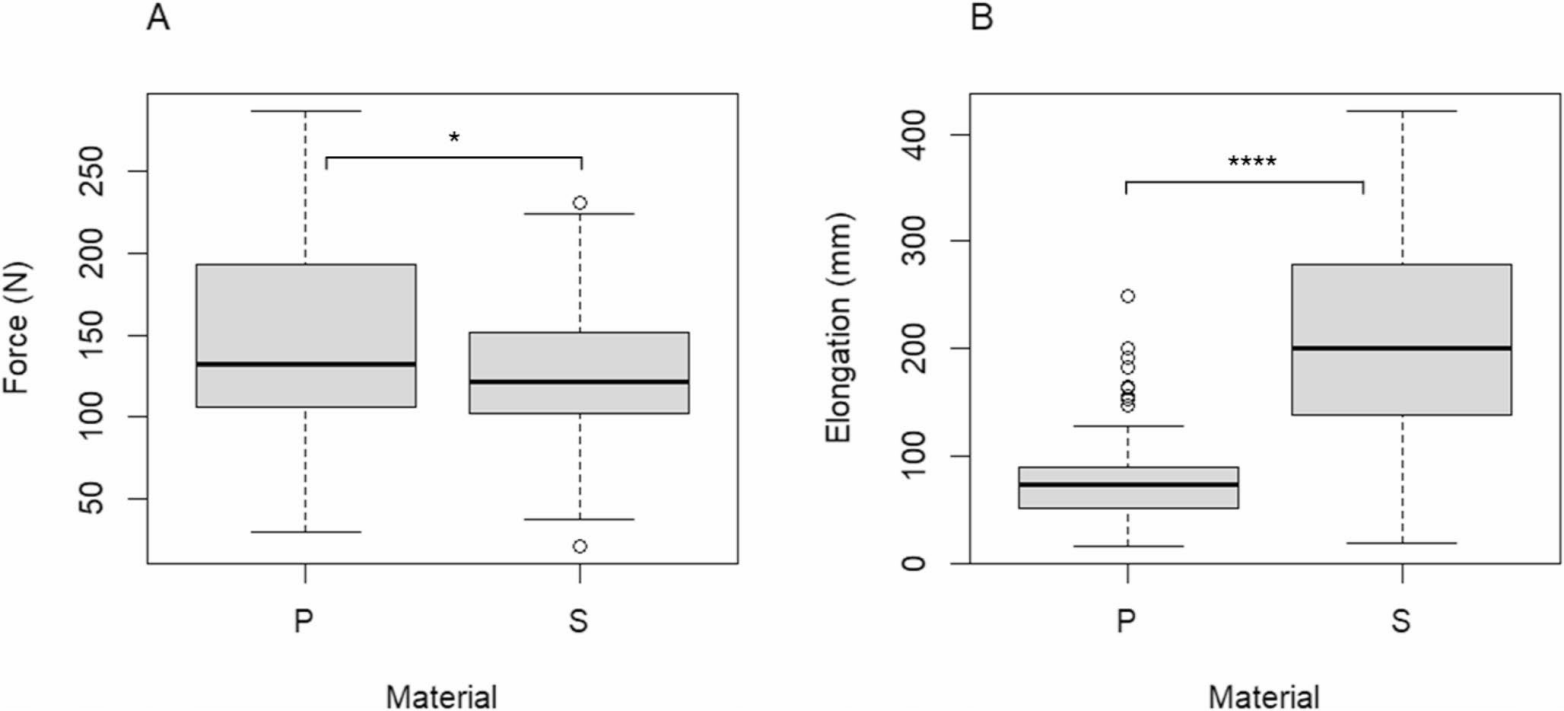
**A** Breaking force in Newton and **B** Elongation in millimeters of two different materials across all tube sizes and suture techniques. P (polyvinyl chloride), S (silicone), * *p* < 0,05, **** *p* < 0.0001

Although silicone tubes (207.3 mm ± 89.4 mm) showed a statistically significantly greater elongation than PVC tubes (76.6 mm ± 33.3 mm; *p* < 0.0001; Fig. 5b), PVC tubes demonstrated a statistically significantly higher pull-out strength (147.0 *N* ± 54.2 mm) compared to silicone tubes (128.5 *N* ± 42.2 mm; *p* = 0.01071; Fig. 5a). The superior biomechanical stability of PVC tubes was particularly pronounced in small (20 Ch) chest tubes using the Modified Johannesburg Technique (Table 2).

### Effect of the tube size

**Fig. 6:**
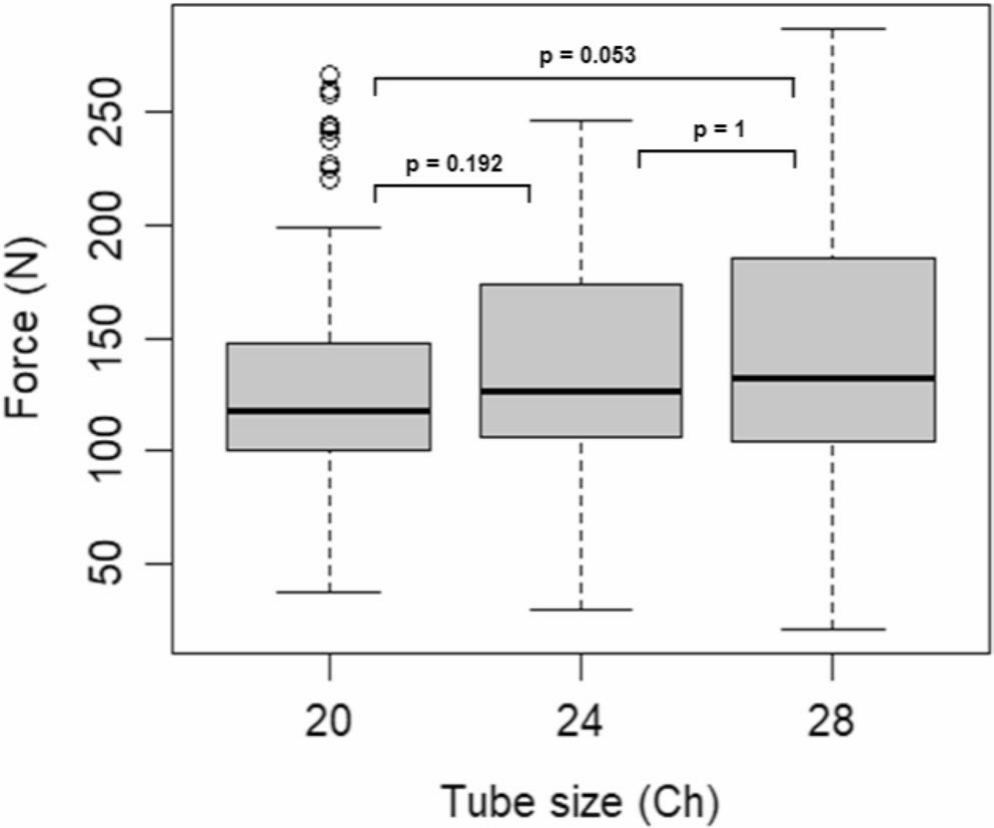
Breaking force in N Newton of three different tube sizes: 20 Ch, 24 Ch and 28 Ch across all materials and suture techniques. Ch: Charrière

Pull-out forces appeared to be influenced by tube size, as indicated by a significant Kruskal-Wallis test (*p* = 0.04461). However, subsequent post-hoc analyses using Dunn’s test with Bonferroni correction did not reveal significant pairwise differences (all *p* > 0.05), suggesting that the overall effect may be modest (Fig. 6).

### Mechanism of failure

**Fig. 7:**
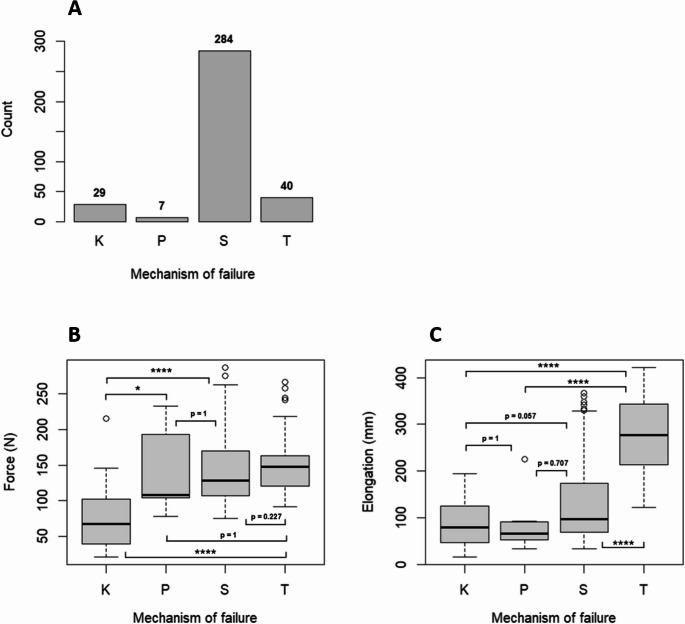
**A** Frequency distribution of the four different failure mechanisms across all groups. **B** Force in Newton of the four different failure mechanisms across all groups. **C** Elongation in millimeters of the four different failure mechanisms across all groups. K: loosening of the knot, P: failure of the pig skin, S: rupture of the suture, T: rupture of the tube, * *p* < 0,05, **** *p* < 0.0001

The most frequent failure mechanism was suture rupture (78.9%), followed by tube rupture (11.1%), knot loosening (8.1%), and skin failure (1.9%) (Fig. 7A). Tube rupture occurred more frequently with the Modified Johannesburg Technique (*n* = 26) than with the Roman Sandal or Purse String techniques (*n* = 7 each; *p* < 0.0001). Silicone tubes ruptured more often than PVC tubes (*n* = 35 vs. *n* = 5; *p* < 0.0001). Smaller tubes also showed higher rupture rates than larger ones (20 Ch: *n* = 31, 24 Ch: *n* = 6, 28 Ch: *n* = 3; *p* < 0.0001). Knot loosening was likewise predominantly observed in small silicone tubes fixed with the Modified Johannesburg Technique. Tube rupture was associated greater elongation (*p* < 0.0001; Fig. 7C).

## Discussion

This study provides a biomechanical comparison of three commonly used chest tube fixation techniques across different tube sizes and materials. The Modified Johannesburg Technique consistently achieved the highest pull-out strength, independent of tube size or material. In contrast, the Roman Sandal and purse-string techniques demonstrated lower mechanical stability, with the Purse String particularly prone to early failure.

The results of the present study are in line with the findings of Ringel et al.: The authors compared the mechanical strength of different fixation techniques for intercostal tubes in a comparable biomechanical setting. They also reported the highest breaking forces with the Modified Johannesburg Technique, followed by the Roman Sandal and purse-string techniques, regardless of suture size used for fixation [14]. Furthermore, Howes et al. compared different suture techniques to secure an ICD by 26 clinicians of various specialties and nationalities. Most of the participants used the Roman Sandal technique with a failure rate of 19.2%, hereas other techniques used showed no failure. These findings imply that suture methods with knots tied close to the skin are more effective, which is also in line with the findings of the present study in a clinical setting [13]. The Modified Johannesburg technique, with its multiple skin anchoring points and superior pull-out resistance, represents the most reliable option, potentially reducing complication rates even under dynamic conditions encountered in combat casualty care or emergency evacuation [11, 14, 15]. Beyond biomechanical performance, practical aspects also influence the clinical suitability of fixation techniques. The Modified Johannesburg Technique, although mechanically superior, may require more time to perform and may be less intuitive for clinicians unfamiliar with its multi-step configuration [11]. In contrast, the Roman Sandal is considered to be faster to apply, easier to learn and more cosmetically favorable, which may explain its widespread use [12, 13, 16]. Moreover, techniques exerting strong skin tension may increase the risk of local skin trauma or patient discomfort, which is especially reported for the Purse-String technique [17, 18]. These procedural and patient-centered factors should be weighed against the mechanical advantages demonstrated in the present study.

The effect of tube size (Ch) was notable: larger tubes generally tolerated higher pull-out forces, especially in silicone tubes, likely due to increased surface contact and suture engagement. PVC tubes exhibited higher absolute forces overall, reflecting the intrinsic material strength [19], but the relative ranking of fixation techniques remained unchanged. While using silicone tubes may increase patient comfort and reduces pain compared to PVC tubes [19, 20], the findings of the present study suggest the use of PVC tubes in case of emergency situations in high-risk environments, including military and prehospital settings. Furthermore, the size of chest tube did not impact the efficacy of drainage, rate of complications including retained hemothorax, need for additional tube drainage, or invasive procedures and the pain felt by patients at the site of insertion in a prospective investigation in 293 patients [21]. Numerous studies have examined the impact of chest tube size on patient comfort and safety, yielding heterogeneous results [22–24]. However, contemporary studies support the use of smaller chest tubes, driven by evidence demonstrating comparable drainage efficacy, reduced patient discomfort, and fewer procedure-related complications [25, 26]. Overall, considering the relatively minor impact of tube size and material compared to suture technique in the present study, the choice of fixation method appears to be the primary determinant of mechanical security. Therefore, the present data should not be interpreted as a recommendation against small-bore tubes in general, but as an indication that tube size may still hold biomechanical relevance in select high-risk settings.

Beyond tube size and material, it remains a matter of debate whether the fixation technique itself is the decisive factor, or rather why certain techniques perform better. The findings of the present study suggest that the suture material represents the weak link in the fixation construct, as suture rupture was by far the most frequent failure mode. Techniques that maximize suture–tube contact and frictional engagement inherently offer greater resistance to pull-out [11, 13, 14]. Thus, the superior stability observed in methods involving multiple wraps and extended suture contact may be a result of the increased frictional resistance rather than the c configuration of the techniques itself. From this perspective, the tensile properties and frictional behavior of the suture material appear to be primary determinants of fixation strength. In contrast, Ringel et al. reported no significant influence of suture caliber on construct stability across different fixation methods [14]. Given that suture rupture accounted for over 75% of failures i the present investigation, future studies should specifically evaluate the impact of suture type, thickness, and material composition on overall fixation stability.

Limitations of this study include the use of porcine cadaveric tissue, which may differ from human thoracic wall properties — particularly in terms of skin elasticity, dermal thickness, and subcutaneous architecture—and may therefore affect the translational applicability of our results. Additionally, the controlled laboratory conditions cannot fully replicate the forces encountered during patient movement or transport. Furthermore, there was no variation in suture sizes or material in the present setting, although suture rupture accounted for the majority of failures. This limits the generalizability of our findings to clinical environments where other commonly used materials such as silk or nylon sutures are routinely applied. Moreover, the experimental setup assessed only vertical pull-out forces; in clinical practice, chest tubes are exposed to multidirectional, repetitive, and occasionally abrupt forces during patient movement or transport, which could influence fixation stability. However, the standardized testing provides valuable comparative biomechanical data.

## Conclusion

The Modified Johannesburg suture offers superior mechanical stability for chest tube fixation compared to Roman Sandal and Purse String techniques, regardless of tube size or material. The primary failure mechanism is suture rupture. The Modified Johannesburg technique should be considered the preferred method in high-risk environments, including military and emergency care, to minimize the risk of tube dislocation and enhance patient safety.

## Supplementary Information

Below is the link to the electronic supplementary material.

Supplementary Material 1

Supplementary Material 2

Supplementary Material 3

## Data Availability

The datasets generated and analyzed are available from the corresponding author on reasonable request.

## Abbreviations

Ch: Charrière
LTF: Load-to-failure
PVC: Polyvinyl chloride
3D: Three-dimensional
ROM: Range of motion
